# Health-promoting lifestyle in mothers with vaginal childbirth and cesarean section in the postpartum period

**DOI:** 10.1186/s12905-024-02984-6

**Published:** 2024-02-26

**Authors:** Motahareh Govahi, Fereshteh Behmanesh, Hemmat Gholinia, Shabnam Omidvar, Hajar Adib-Rad

**Affiliations:** 1 Student Research Committee, Babol University of Medical Sciences, Babol, I.R. of Iran; 2 Social Determinants of Health Research Center, Health Research Institute, Babol University of Medical Sciences, Babol, I.R. of Iran; 3 Health research Institute, Babol University of Medical Sciences, Babol, I.R. of Iran

**Keywords:** Lifestyle, Vaginal childbirth, Cesarean section, Mothers, Postpartum

## Abstract

**Background:**

In the postpartum period, there are numerous changes in the physical and psychological dimensions of women, which reduce the quality of life of women. The aim of this study was to compare the health-promoting lifestyle of mothers with vaginal delivery and cesarean delivery in the postpartum period.

**Methods:**

This cross-sectional study was conducted on 77 pregnant women who had delivered vaginal or by cesarean section at Shohadaye Behshahr Hospital and were selected based on inclusion criteria. If the women were willing to participate in the study, a demographic questionnaire was completed, and the telephone numbers of the subjects were recorded so that the Porsline health-promoting lifestyle questionnaire could be sent to them. Data were analyzed with SPSS 22 using the T test, chi-square test and Repeated Measure ANOVA.

**Results:**

There was no difference in the average score of health-promoting lifestyle and its dimensions between the two groups of vaginal delivery and cesarean section at two and six weeks after delivery. However, in both groups, the total score of health-promoting lifestyle decreased significantly over time (*P* < 0.001).

**Conclusions:**

There was no difference in health-promoting lifestyle between mothers with vaginal and mothers with cesarean delivery at two weeks and six weeks after delivery. This requires more attention from policy makers to make vaginal childbirth more convenient, and by reducing complications after vaginal childbirth, they can improve women’s healthy lifestyles and, in turn, families. Also, it seems that the other variables apart from the method of delivery should be considered, and it is necessary to distinguish these variables such as routine episiotomy in order to prevent the decrease in the level of health-promoting behaviors among women during puerperium period.

**Supplementary Information:**

The online version contains supplementary material available at 10.1186/s12905-024-02984-6.

## Background

A health-promoting lifestyle is one of the most important determinants of health [1–3] and includes a set of actions taken to maintain and improve the health of individuals and society. These behaviors include the six dimensions of nutrition, physical activity, stress management, interpersonal relationships, spiritual growth, and health responsibility [4–6]. According to a World Health Organization report, 40–50% of deaths in developing countries are due to behaviors to maintain and promote health [7].

As one of the pillars of the family, women play an important role in family health, and performing this role requires physical and mental health [8] A health-promoting lifestyle is very important for women of childbearing age when health problems such as diseases related to pregnancy, childbirth, and breastfeeding occur [9, 10].

In the postpartum period, there are numerous physical and psychological changes that affect women’s quality of life during this time [11–13]. Fatigue, insomnia, breast tenderness, physical pain, constipation, and sexual dysfunction in the postpartum period are largely related to the type of delivery [14–17].

The results of the study by Kohler et al. (2018) showed that the quality of life of women with vaginal delivery even with episiotomy was higher than that of women with cesarean delivery [18]. The study by Jorfi et al. (2015) also indicated that the mode of delivery was related to a health-promoting lifestyle, and cesarean section had the most negative impact on health-promoting behaviors [19]. However, in the study by Radnia et al. (2017), no significant association was found between the mode of delivery and quality of life dimensions [20].

Women who have delivered by cesarean section are more likely to experience problems such as fatigue, headache, urinary tract infections, anemia, abnormal bleeding, breastfeeding problems, and abdominal pain than women who have delivered vaginally [21]. Therefore, women who undergo cesarean delivery may not be able to practice some health-promoting behaviors. On the other hand, quality of life after delivery is compromised when vaginal delivery is accompanied by an episiotomy. In a qualitative study, women’s experiences of vaginal childbirth with an episiotomy suggested that the pain caused by the episiotomy affected women’s daily lives for weeks [22].

Since maternal health is directly related to the health of the family and society, it is very important to study the health-promoting lifestyle of mothers in the first six months after giving birth. Therefore, the aim of this study was to compare the health-promoting lifestyle of mothers with vaginal delivery and cesarean delivery in the postpartum period.

## Methods

This cross-sectional study was conducted in Shohadaye Behshahr Hospital (Hospital for Women) in 2021 after approval by the Ethics Committee of Babol University of Medical Sciences under the number IR.MUBABOL.HRI.REC.1400.058. The recruitment, exposure, follow-up and data collection periods lasted from November 2021 to September 2022. In this hospital, episiotomy is performed in 70.74% of normal parturients, and the rate of episiotomy in primiparous women is almost 100%.

The samples studied were women who had given birth in the postpartum ward of Shohadaye Behshahr Hospital and were selected based on inclusion criteria. Study participation criteria were as follows: literacy, willingness to participate in the study, no maternal or infant hospitalization, no high-risk pregnancy, no history of systemic disease, and no known mental illness. The exclusion criteria were as follows: failure of the mother to continue to cooperate and failure to answer more than 10% of the questions in the questionnaire.

The number of samples using the information from the previous study [23] with a mean of 133.7, a standard deviation of 3 for health-promoting lifestyle variable in the six weeks after birth, a power of 80, an alpha value of 0.05, and a 10% dropout was considered as 40 subjects in each group and a total number of 80 subjects in the study.

After the objectives of the study were discussed, written informed consent was obtained, the demographic questionnaire was completed, the telephone numbers of the women were recorded, and the Porsline health-promoting lifestyle questionnaire was sent to them via cellphone and completed two weeks and six weeks after delivery.

The demographic questionnaire included the variables of age, age of spouse, affordability of living expenses, occupation of the woman and man, educational level of the woman and man, mode of delivery, number of pregnancies, number of abortions and unwanted pregnancies.

The health-promoting lifestyle questionnaire consisted of 52 questions. In this questionnaire, six dimensions of diet, exercise, responsibility for health, stress management, interpersonal support, and self-actualization are assessed. The response spectrum is Likert type, and the score for each option is never [1], sometimes [2], often [3], and always [4].

The score for the health-promoting lifestyle and the score for the behavioral dimensions are obtained by using the average responses for all 52 questions and for each dimension. To obtain the scores for each dimension, the total scores of the questions on that dimension are added, and to obtain the total score of the questionnaire, the total scores of all questions are added. The score of the questionnaire ranges from 52 to 208.

Higher values indicate better health-promoting behaviors. In the study of Mohammadi-Zeidi et al. (2010), the validity of the questionnaire was confirmed [24].

After obtaining information using the Porsline questionnaire from mothers two and six weeks after delivery, the data were analyzed using SPSS 22 through the independent t test, chi-square test and Repeated Measure ANOVA. The independent t-test was used for comparison of quantitative demographic variables (age, age of spouse, number of pregnancies and number of abortions). The Repeated Measure ANOVA was used to compare the mean health-promoting lifestyle scores and its dimensions at 2 weeks and 6 weeks after delivery in mothers with vaginal and cesarean delivery.

## Results

**Fig. 1:**
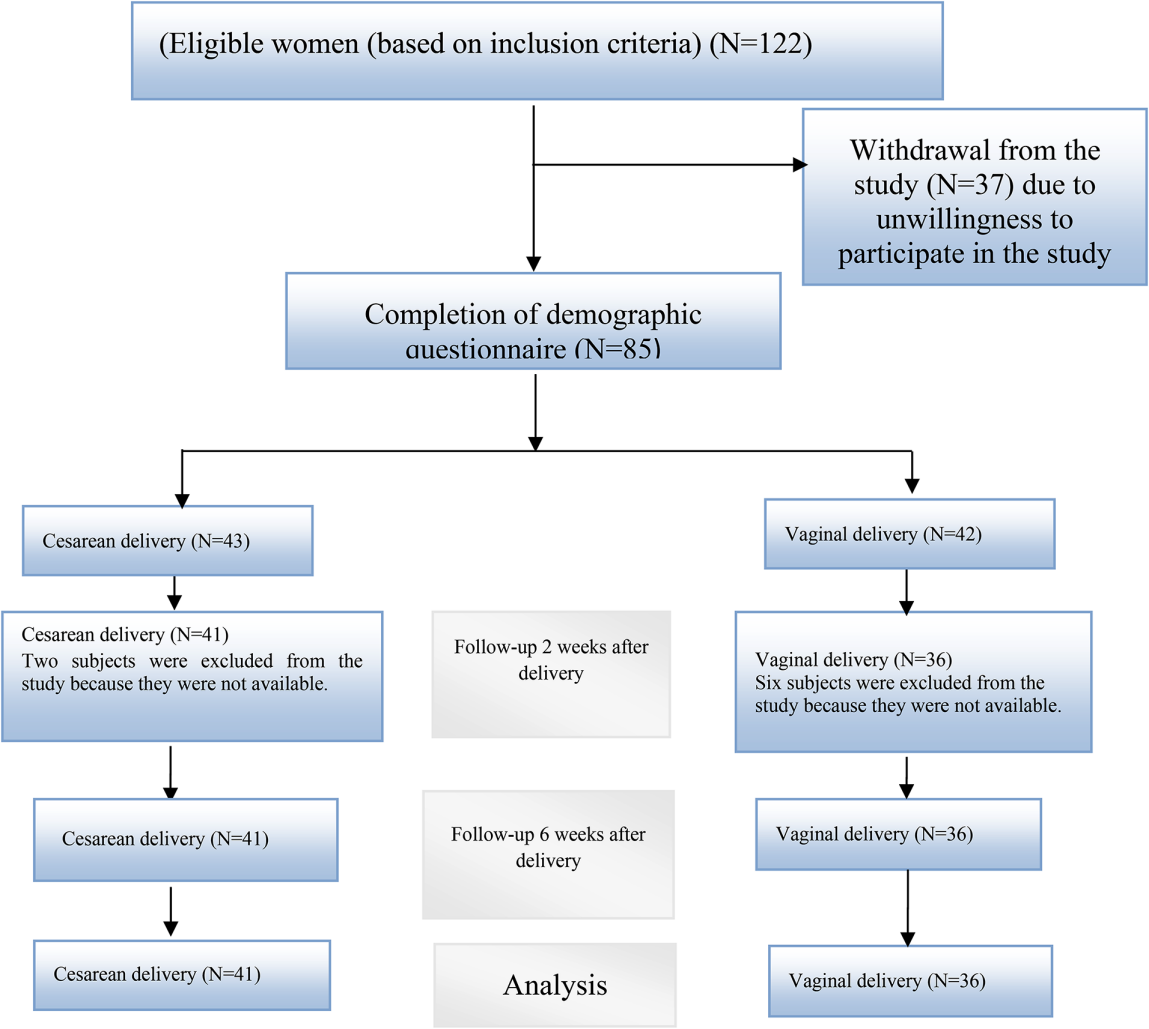
Sampling diagram

A total of 77 patients were analyzed in the present study, of whom 41 delivered by cesarean section and 36 by vaginal means (Fig. 1). The mean age of mothers with cesarean delivery was 28.4 ± 6.06 years, and the mean age of mothers with vaginal delivery was 27.4 ± 6.2 years. Most women in both groups had diplomas, and most women in both groups were housewives. Of the 36 women who had a normal delivery, 32 had an episiotomy, and 3 had a first-degree laceration. Some demographic information about the patients studied is shown in Table 1.

**Table 1:** Demographic and midwifery information of the studied women

Groups	Variables	(NVD)	(C/S)
		Frequency (%)	Frequency (%)
Education level	High school	8(22.2)	5(12.2)
	Diploma	17(47.2)	19(46.3)
	University	11(30.6)	17(41.5)
place of residence	Village	15(41.7)	14(34.1)
	City	21(58.3)	27(65.9)
Job	Unemployed	30(83.3)	30(73.2)
	Employed	6(16.7)	11(26.9)
Number of pregnancies	1	13 (16.9)	19(24.7)
	> 1	23 (29.9)	22 (28.6)
Number of abortions	1	27 (35.1)	31 (40.3)
	> 1	9 (11.7)	10 (13.0)
Unwanted pregnancy	NO	33 (42.9)	37 (48.1)
	Yes	7 (3.9)	4 (5.2)

Comparison of mean health-promoting lifestyle scores at 2 weeks (NVD: 132.64 ± 23.99 vs. C/S: 134.61 ± 19.18) and 6 weeks (NVD: 129.22 ± 22.26 vs. C/S: 129.07 ± 22.64) after delivery in mothers with vaginal and cesarean delivery showed that mean lifestyle scores in the two groups decreased over time (*P* < 0.001). However, the group effect was not significant (*P* = 0.848, effect size = 0.0004) (Table 2).

**Table 2:** Comparison of changes in the mean score of health-promoting lifestyle two weeks and six weeks after delivery in mothers with vaginal delivery and cesarean delivery

Variables	Time	second week	Sixth week	*P _time_	*P _group_	Effect size
Self-Actualization	C/S	33.95 ± 5.87	31.98 ± 6.66	0.001	0.106	0.034
	NVD	31.69 ± 6.52	29.86 ± 6.41			
Health Responsibility	C/S	32.27 ± 7.00	30.51 ± 6.96	0.084	0.536	0.005
	NVD	32.64 ± 8.38	32.11 ± 7.72			
Interpersonal Support	C/S	22.24 ± 4.18	21.85 ± 4.91	0.106	0.515	0.006
	NVD	23.25 ± 4.58	22.06 ± 4.54			
Stress Management	C/S	11.54 ± 2.66	11.00 ± 2.63	0.297	0.538	0.005
	NVD	11.53 ± 2.91	11.39 ± 2.49			
Exercise	C/S	12.93 ± 4.69	13.27 ± 4.24	0.773	0.835	0.0003
	NVD	13.31 ± 4.67	13.19 ± 4.68			
Nutrition	C/S	21.68 ± 4.33	20.46 ± 4.69	0.349	0.508	0.006
	NVD	20.22 ± 4.91	20.61 ± 5.02			
Total health-promoting lifestyle profile	C/S	134.61 ± 19.18	129.07 ± 22.64	0.008	0.848	0.0004
	NVD	132.64 ± 23.99	129.22 ± 22.26			

* Repeated Measure ANOVA

Mean scores on the dimensions of self-actualization, responsibility for health, interpersonal support, stress management, exercise, and diet also decreased over time in both groups. But were not significant. There was significant difference in Self-Actualization dimension only (*P* < 0.001). Also, there was no difference between the two groups in the dimensions of health-promoting lifestyle two weeks and six weeks later (*P* > 0.05).

## Discussion

The aim of the current study was to compare the health-promoting lifestyle habits of mothers with vaginal delivery and cesarean delivery in the postpartum period. According to the results of this study, there was no difference in the mean score of health-promoting lifestyle and its dimensions between the two groups at two weeks and six weeks.

In the study by Radnia et al. (2018), there was no significant association between mode of delivery and quality of life dimensions, which is consistent with the present study. Of course, the study by Radnia et al. (2018) used the quality of life questionnaire, which is different from the instrument used in the present study [20].

In the study by Nikpour et al., eight weeks after delivery, the mean physical and mental quality of life scores were significantly higher in the vaginal delivery group than in the cesarean delivery group. However, there was no significant difference between the two groups in overall quality of life scores [25]. The results of the study by Trivino-Juarez et al. (2016) on quality of life at the sixth week and six months postpartum in women with vaginal delivery and cesarean delivery demonstrated no difference between the two groups, and the results of the present study were consistent with their study [26].

However, in the study by Jorfi et al. (2015), a significant association was found between the mode of delivery (vaginal and cesarean) and health-promoting behaviors, which contradicts the present study. In the study, women who underwent cesarean section had poorer lifestyle behaviors than women who delivered naturally [19]. One of the reasons for the difference between the current study and the study by Jorfi et al. is probably that in the study by Jorfi et al., breastfeeding women who gave birth less than 2 months after delivery were excluded from the study. However, in the present study, subjects completed the health-promoting lifestyle questionnaire less than two months after delivery. It seems that the time of completing the questionnaires and the physical condition of the women at the time of completing the questionnaires have an influence on the results.

Another finding of the present study was that the total value of health-promoting lifestyle and its dimensions decreased six weeks after delivery in mothers with vaginal delivery and cesarean section compared with two weeks after delivery. However, the results of the study by Trivino-Juarez et al. (2016) indicated that health-related quality of life improved between the sixth week and sixth month after delivery in both vaginal and cesarean deliveries, which contradicts the present study. The study by Trivino-Juarez et al. compared six weeks after delivery with six months after delivery [26], whereas in the present study, the follow-up period was different.

In Iran, fathers are granted a two-week leave after the birth of a child so that they can be with their wives and children and support them. The mother is also supported by her family or husband during this time, and families help the new mother with housework and caring for the baby. Usually, during the first two weeks after birth, the mother’s tasks are limited to breastfeeding the baby and personal hygiene.

However, after this period, when the stitches caused by cesarean section and vaginal delivery are almost healed and the mother is able to manage the household, the father’s two-week leave ends and daily support from the wife must cease. On the other hand, the relatives leave the mother alone with the newborn and the many household chores. One of the most important reasons for the decline in health-promoting behavior scores among the mothers in the present study during the two- to six-week postpartum period is probably the loss of support from the spouse and family members.

Another case that can be used as a rationale for the reduction in health-promoting behavior scores among mothers with vaginal childbirth was the high rate of episiotomy in primiparous mothers in this study. In a qualitative study, He et al. (2019) revealed that the pain caused by episiotomy affected women’s daily lives in different ways. Among these, breastfeeding and defecation problems were mentioned by most participants. Episiotomy pain interferes with breastfeeding. Normally, women like to breastfeed their babies while sitting, but when it is painful, they have problems breastfeeding [22].

Psychological shadow was one of the issues He et al. (2019) achieved in their qualitative study. Unfavorable sex life and less confidence for later vaginal delivery were the classes from which the theme of psychological shadow was derived [22]. All of these factors may influence the value of health-promoting behaviors in women with vaginal childbirth with episiotomy.

Even in Iran, some hospitals have been considered mother- and baby-friendly hospitals, and these hospitals are running physiologic deliveries. Additionally, all pregnant mothers in the clinics and hospitals attend preparation programs during pregnancy that reduce the chances of laceration during delivery. Therefore, it is suggested that based on the guidelines of the Iranian Ministry of Health and Medicine regarding the promotion of vaginal delivery, episiotomy even in primiparous women should be based on the indication and not routine.

The limitations of the present study were the lack of assessment of some social and psychological variables that influence women’s health-promoting behaviors, such as mental health, family economic status, and women’s independence. On the other hand, because this study was conducted in one hospital and in one geographic area, the generalizability of the results should be discussed with caution.

It is suggested that episiotomy be removed from routine practice and limited to primiparous mothers who have a very short perineum. It also suggested a study with more sample size for the future study.

## Conclusions

The health-promoting behaviors two and six weeks after delivery did not exhibit significant difference among women with different mode of delivery. According to the results, it seems that the other factors apart from the method of delivery should be considered, and it is necessary to identify these factors such as routine episiotomy in order to prevent the decrease in the level of health-promoting behaviors among women during puerperium period.

### Electronic supplementary material

Below is the link to the electronic supplementary material.

**Supplementary Material 1:** LIFESTYLE PROFILE II

**Supplementary Material 2:** HEALTH-PROMOTING LIFESTYLE PROFILE II: Scoring Instructions

**Supplementary Material 3:** Questionnaire of personal and social characteristics

**Supplementary Material 4:** STROBE Statement—checklist

## Data Availability

The datasets used during the current study are available from the corresponding author upon reasonable request.

## Abbreviations

SPSS: Statistical Package for the Social Sciences
ANOVA: Analysis Of Variance
NVD: Natural Vaginal Delivery
C/S: Cesarean Section
