# Supplementary material for: Health-promoting lifestyle in mothers with vaginal childbirth and cesarean section in the postpartum period
Source: BMC Womens Health. 2024 Feb 26;24:146. doi: 10.1186/s12905-024-02984-6 (PMC10898097; doi:10.1186/s12905-024-02984-6)
Supplement: Supplementary file 2 — Supplementary Material 2: HEALTH-PROMOTING LIFESTYLE PROFILE II: Scoring Instructions [file 12905_2024_2984_MOESM2_ESM.pdf]

HEALTH-PROMOTING LIFESTYLE PROFILE II

Scoring Instructions

Items are scored as			
	Never (N)	=	1
	Sometimes (S)	=	2
	Often (O)	=	3
	Routinely (R)	=	4

A score for overall health-promoting lifestyle is obtained by calculating a mean of the individual's responses to all 52 items; six subscale scores are obtained similarly by calculating a mean of the responses to subscale items. The use of means rather than sums of scale items is recommended to retain the 1 to 4 metric of item responses and to allow meaningful comparisons of scores across subscales. The items included on each scale are as follows:

Health-Promoting Lifestyle	1 to 52
Health Responsibility	3, 9, 15, 21, 27, 33, 39, 45, 51
Physical Activity	4, 10, 16, 22, 28, 34, 40, 46
Nutrition	2, 8, 14, 20, 26, 32, 38, 44, 50
Spiritual Growth	6, 12, 18, 24, 30, 36, 42, 48, 52
Interpersonal Relations	1, 7, 13, 19, 25, 31, 37, 43, 49
Stress Management	5, 11, 17, 23, 29, 35, 41, 47

3/95: snw
