# Supplementary material for: Health-promoting lifestyle in mothers with vaginal childbirth and cesarean section in the postpartum period
Source: BMC Womens Health. 2024 Feb 26;24:146. doi: 10.1186/s12905-024-02984-6 (PMC10898097; doi:10.1186/s12905-024-02984-6)
Supplement: Supplementary file 3 — Supplementary Material 3: Questionnaire of personal and social characteristics [file 12905_2024_2984_MOESM3_ESM.docx]

**Questionnaire of personal and social characteristics**

Code: ..................... Contact number: …………………………..

1. Age? ......................
2. Age of spouse? ..........
3. Occupation of the woman?

1) Unemployed 2) Employed

1. Occupation of the man?

1) Unemployed 2. Farmer 3. Worker 4. Employee 5. Freelance 6.Specialist (engineering, medicine, judgment, etc.) 7. Manager 8. Student

1. Educational level of the woman?

1) High school 2) Diploma 3) University

1. Educational level of the man?

1) High school 2) Diploma 3) University

1. Place of residence?

1) Village 2) City

1. Mode of delivery?

1) Natural with tools 2) Natural without tools 3) Cesarean section

1. Number of pregnancy? ...........................
2. Number of abortions? ……………….
3. Was this pregnancy unwanted?
4. No 2)Yes
