# Supplementary material for: Health-promoting lifestyle in mothers with vaginal childbirth and cesarean section in the postpartum period
Source: BMC Womens Health. 2024 Feb 26;24:146. doi: 10.1186/s12905-024-02984-6 (PMC10898097; doi:10.1186/s12905-024-02984-6)
Supplement: Supplementary file 4 — Supplementary Material 4: STROBE Statement—checklist [file 12905_2024_2984_MOESM4_ESM.docx]

STROBE Statement—checklist: **Health-promoting lifestyle in mothers with natural childbirth and cesarean section in the postpartum period**

|  | Item No. | Recommendation | Page  No. | Relevant text from manuscript |
| --- | --- | --- | --- | --- |
| **Title and abstract** | 1 | (*a*) Indicate the study’s design with a commonly used term in the title or the abstract | 1 |  |
|  |  | (*b*) Provide in the abstract an informative and balanced summary of what was done and what was found | 2 | cross-sectional study |
| Introduction | | | |  |
| Background/rationale | 2 | Explain the scientific background and rationale for the investigation being reported | 3 |  |
| Objectives | 3 | State specific objectives, including any prespecified hypotheses | 3 | The aim of this study was to compare the health-promoting lifestyle habits of mothers with natural delivery and cesarean delivery in the postpartum period. |
| Methods | | | |  |
| Study design | 4 | Present key elements of study design early in the paper | 3 | cross-sectional study |
| Setting | 5 | Describe the setting, locations, and relevant dates, including periods of recruitment, exposure, follow-up, and data collection | 3 | Shohadaye Behshahr Hospital, The recruitment, exposure, follow-up and data collection periods lasted from November 2021 to September 2022. |
| Participants | 6 | (*a*) *Cohort study*—Give the eligibility criteria, and the sources and methods of selection of participants. Describe methods of follow-up  *Case-control study*—Give the eligibility criteria, and the sources and methods of case ascertainment and control selection. Give the rationale for the choice of cases and controls  *Cross-sectional study*—Give the eligibility criteria, and the sources and methods of selection of participants | 3 & 4 | The samples studied were women who had given birth in the postpartum ward of Shohadaye Behshahr Hospital and were selected by convenience sampling method. Study participation criteria were: literacy, willingness to participate in the study, no maternal or infant hospitalization, no high-risk pregnancy, no history of systemic disease, and no known mental illness and exclusion criteria were: Failure of the mother to continue to cooperate and failure to answer more than 10% of the questions in the questionnaire. |
| Variables | 7 | Clearly define all outcomes, exposures, predictors, potential confounders, and effect modifiers. Give diagnostic criteria, if applicable | 4 | age, age of spouse, affordability of living expenses, occupation of the woman and man, educational level of the woman and man, mode of delivery, and number of children.and health-promoting lifestyle |
| Data sources/ measurement | 8* | For each variable of interest, give sources of data and details of methods of assessment (measurement). Describe comparability of assessment methods if there is more than one group | *N/A* |  |
| Bias | 9 | Describe any efforts to address potential sources of bias | - |  |
| Study size | 10 | Explain how the study size was arrived at | 4 | The number of samples using the information from the previous study (23) with a mean of 133.7, a standard deviation of 3 for health-promoting lifestyle variable in the six weeks after birth, a power of 80, an alpha value of 0.05, and a 10% dropout was considered as 40 subjects in each group and a total number of 80 subjects in the study. |

Continued on next page

| Quantitative variables | 11 | Explain how quantitative variables were handled in the analyses. If applicable, describe which groupings were chosen and why | 4 |  |
| --- | --- | --- | --- | --- |
| Statistical methods | 12 | (*a*) Describe all statistical methods, including those used to control for confounding | 4 | The data were analyzed using SPSS 22 through the independent t-test, paired t-test, and ANCOVA. |
|  |  | (*b*) Describe any methods used to examine subgroups and interactions | - |  |
|  |  | (*c*) Explain how missing data were addressed | - |  |
|  |  | (*d*) *Cohort study*—If applicable, explain how loss to follow-up was addressed  *Case-control study*—If applicable, explain how matching of cases and controls was addressed  *Cross-sectional study*—If applicable, describe analytical methods taking account of sampling strategy | 4 | The data were analyzed using SPSS 22 through the independent t-test, paired t-test, and ANCOVA. |
|  |  | (*e*) Describe any sensitivity analyses | - |  |
| Results | | | | |
| Participants | 13* | (a) Report numbers of individuals at each stage of study—eg numbers potentially eligible, examined for eligibility, confirmed eligible, included in the study, completing follow-up, and analysed | 4 and 12 | By using a flow diagram(Figure 1) |
|  |  | (b) Give reasons for non-participation at each stage | 12 | By using a flow diagram (Figure 1) |
|  |  | (c) Consider use of a flow diagram | 12 | By using a flow diagram(Figure 1) |
| Descriptive data | 14* | (a) Give characteristics of study participants (eg demographic, clinical, social) and information on exposures and potential confounders | 4 and 10 | Table 1 |
|  |  | (b) Indicate number of participants with missing data for each variable of interest | 12 | Figure 1 |
|  |  | (c) *Cohort study*—Summarise follow-up time (eg, average and total amount) | - | - |
| Outcome data | 15* | *Cohort study*—Report numbers of outcome events or summary measures over time | *-* | *-* |
|  |  | *Case-control study—*Report numbers in each exposure category, or summary measures of exposure | *-* | *-* |
|  |  | *Cross-sectional study—*Report numbers of outcome events or summary measures | *4 & 5* |  |
| Main results | 16 | (*a*) Give unadjusted estimates and, if applicable, confounder-adjusted estimates and their precision (eg, 95% confidence interval). Make clear which confounders were adjusted for and why they were included | N/A |  |
|  |  | (*b*) Report category boundaries when continuous variables were categorized | 4 & 5 |  |
|  |  | (*c*) If relevant, consider translating estimates of relative risk into absolute risk for a meaningful time period | N/A |  |

Continued on next page

| Other analyses | 17 | Report other analyses done—eg analyses of subgroups and interactions, and sensitivity analyses | 4 & 5 |  |
| --- | --- | --- | --- | --- |
| Discussion | | | | |
| Key results | 18 | Summarise key results with reference to study objectives | 5 | According to the results of this study, there was no difference in the mean score of health-promoting lifestyle and its dimensions between the two groups at the time two weeks and six weeks later. |
| Limitations | 19 | Discuss limitations of the study, taking into account sources of potential bias or imprecision. Discuss both direction and magnitude of any potential bias | 6 | The limitations of the present study were the impossibility of random sampling and the lack of assessment of some social and psychological variables that influence women's health-promoting behaviors, such as: mental health, family economic status, and women's independence. On the other hand, because this study was conducted in one hospital and in one geographic area, the generalizability of the results should be discussed with caution. |
| Interpretation | 20 | Give a cautious overall interpretation of results considering objectives, limitations, multiplicity of analyses, results from similar studies, and other relevant evidence | 5 & 6 |  |
| Generalisability | 21 | Discuss the generalisability (external validity) of the study results | 6 | Because this study was conducted in one hospital and in one geographic area, the generalizability of the results should be discussed with caution. |
| Other information | |  | | |
| Funding | 22 | Give the source of funding and the role of the funders for the present study and, if applicable, for the original study on which the present article is based | 7 | This study was funded and supported by Babol University of Medical Sciences. (Grant No. 724133900) |

*Give information separately for cases and controls in case-control studies and, if applicable, for exposed and unexposed groups in cohort and cross-sectional studies.

**Note:** An Explanation and Elaboration article discusses each checklist item and gives methodological background and published examples of transparent reporting. The STROBE checklist is best used in conjunction with this article (freely available on the Web sites of PLoS Medicine at http://www.plosmedicine.org/, Annals of Internal Medicine at http://www.annals.org/, and Epidemiology at http://www.epidem.com/). Information on the STROBE Initiative is available at www.strobe-statement.org.
